# Beer-gut microbiome alliance: a discussion of beer-mediated immunomodulation via the gut microbiome

**DOI:** 10.3389/fnut.2023.1186927

**Published:** 2023-07-25

**Authors:** Silu Zhang, Shuo Jin, Cui Zhang, Shumin Hu, Huajun Li

**Affiliations:** ^1^Department of Microecology, Dalian Medical University, Dalian, China; ^2^State Key Laboratory of Biological Fermentation Engineering of Beer, Tsingtao Brewery Co. Ltd., Qingdao, China

**Keywords:** beer, nutrition, gut microbiome, food immunomodulatory, mucosal barrier, antioxidant

## Abstract

As a long-established fermented beverage, beer is rich in many essential amino acids, vitamins, trace elements, and bioactive substances that are involved in the regulation of many human physiological functions. The polyphenols in the malt and hops of beer are also important active compounds that interact in both directions with the gut microbiome. This review summarizes the mechanisms by which polyphenols, fiber, and other beneficial components of beer are fermentatively broken down by the intestinal microbiome to initiate the mucosal immune barrier and thus participate in immune regulation. Beer degradation products have anti-inflammatory, anticoagulant, antioxidant, and glucolipid metabolism-modulating potential. We have categorized and summarized reported data on changes in disease indicators and *in vivo* gut microbiota abundance following alcoholic and non-alcoholic beer consumption. The positive effects of bioactive substances in beer in cancer prevention, reduction of cardiovascular events, and modulation of metabolic syndrome make it one of the candidates for microecological modulators.

## 1. Introduction

Beer, also known as “liquid bread,” is the oldest alcoholic beverage in human history, recorded by the Babylonians as early as 6,000 BC using clay tablets. At the same time, beer is the most widely produced and consumed beverage globally. It is second only to water and tea in terms of total consumption. Archeological research has found evidence of beer consumption in China dating back as far as 9,000 years (1). In response to consumer demand for new taste, smell, and visual stimuli, and for a variety of beer types, including non-alcoholic beers, beers produced by producers who add fruits, spices, vegetables, and natural foods to the fermentation process are becoming very popular around the world (2). At the same time, the health properties of beer that cannot be ignored are also gaining attention. It has been reported that beer consumption has a regulatory effect on various physiological functions of the human body. Moderate consumption of beer helps in preventing arteriosclerosis (3) and heart disease (4), inhibits cancer (5), and improves blood circulation and immune function (6). Beer has also been shown to have antioxidant and anti-aging effects (7), promote estrogen production (8), reduce radiation damage (9), and help prevent cardiovascular events (10).

Beer is brewed from malt, hops, yeast, and brewing water as well as starchy and sugary auxiliary ingredients through liquid pasting, saccharification, and liquid fermentation (11). Like yogurt, wine, cider, and many other fermented beverages, beer is rich in many nutrients. It contains many essential amino acids, vitamins, trace elements, and biologically active substances such as polyphenols and flavonoids. Beer also contains many minerals such as calcium, magnesium, zinc, copper, selenium, and iron. Beta-glucan and arabinose-oligosaccharides stored in malting barley also make beer a source of dietary fiber (12). In addition, the polyphenols in the malt and hops of beer are essential active compounds with antioxidant activity that act synergistically with dietary components. The amino acids, vitamins, inorganic salts, and low molecular sugars in beer are quickly digested and absorbed in the small intestine. Polyphenols that are not hydrolyzed by the small intestine reach the colon and are metabolized by the body’s microbiota (13).

Nutrients in the host’s diet can influence the growth of the gut microbiome. In the meantime, specific dietary components can stimulate the gut microbiome to secrete metabolites that affect the host’s physiological status. This ‘super-organ’ residing in the gut is an essential medium for the body’s intake of nutrients. It is involved in the absorption and metabolism of nutrients, strengthens the integrity of the gut, prevents the spread of pathogens, promotes immune tolerance to antigens, and regulates host immunity, thereby directly influencing human health and disease (14).

This review focuses on the potential mechanisms by which polyphenols and other beneficial components in beer exert prebiotic effects, interact with the gut microbiome, and participate in immune regulation, thus exploring beer’s immune utility and application prospects as a health food.

## 2. Beer has the effect of improving immune function

Drinking alcohol, which is a regular habit for many people, is controversial in terms of its effects on human health. It is well known that beer, as an alcoholic beverage, can induce serious tissue damage and organ lesions such as increased intestinal inflammation, endotoxemia, and alcoholic liver disease if consumed inappropriately or in excess (15). It can also amplify the damage caused by cardiac insufficiency and depression in patients, adolescents, and pregnant women (16).

**Figure 1:**
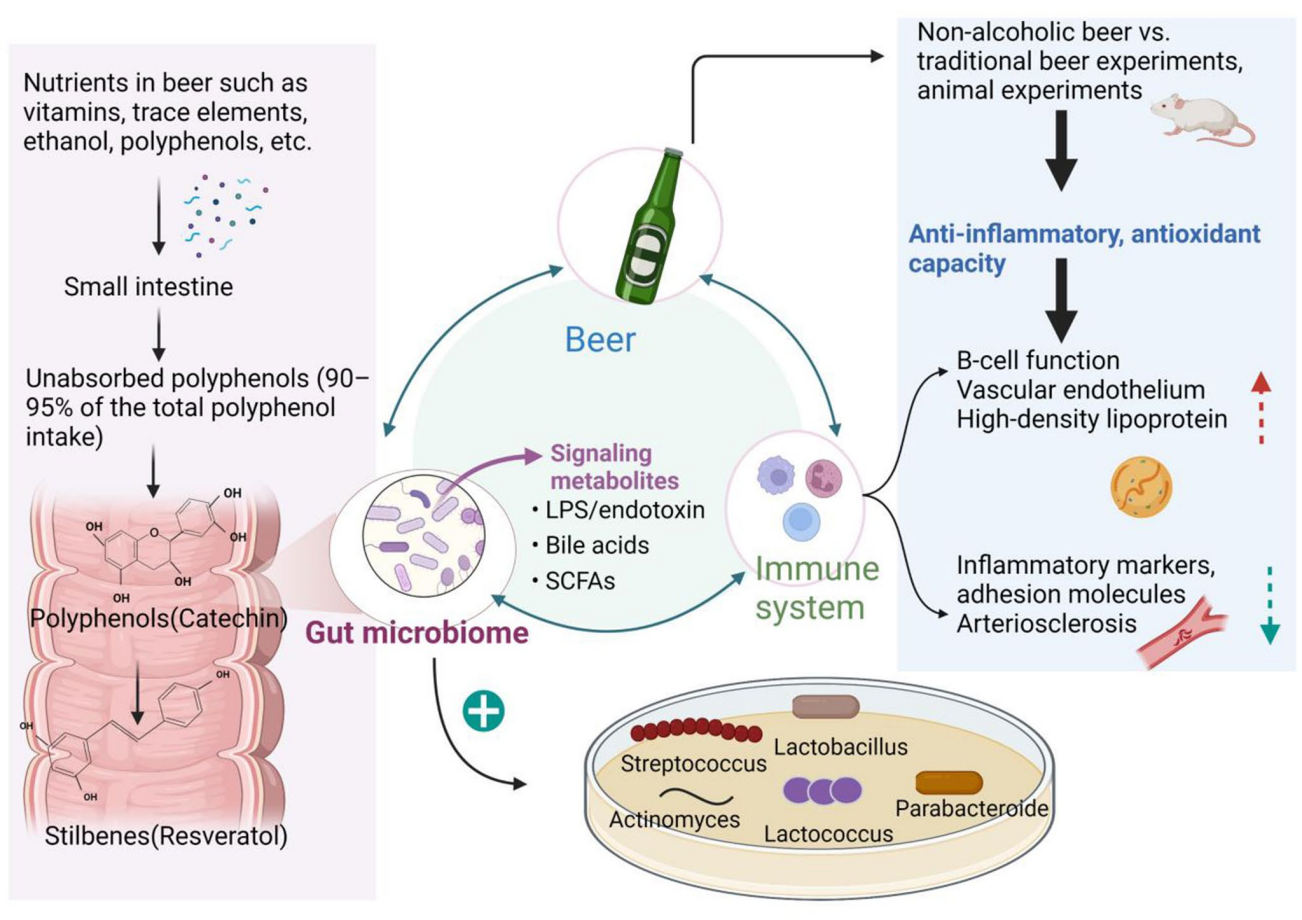
Relationship between beer, immunity, and the gut microbiome. When beer is consumed in moderation, the phenols and other nutrients it contains are fermented and broken down by the microbial community that resides in the outer mucosal layer of the gut. This miraculous digestive process produces a wealth of metabolites that, through the interaction of multiple microbes in the inner mucus, in turn, promote the growth of beneficial flora that exert a range of anti-inflammatory, antioxidant, and immunomodulatory effects, creating a virtuous cycle. This gives beer, a fermented beverage, a place in the improvement of cardiovascular disease, obesity, diabetes, neurodegenerative diseases, cancer, non-alcoholic fatty liver disease, and the prevention of infections.

However, when alcohol consumption is controlled within safe limits, the nutrients in beer and the combined effects on the gut microbiome have a positive effect on the regulation of human immune function (15) (Figure 1). Beer reduces leukocyte adhesion molecules and biological risk markers of inflammation and increases plasma antioxidant capacity, whereas ethanol alone does not (17, 18). We will describe the effect of beer intake on organismal immunity through the aspect of conventional, and non-alcoholic beer intake in humans, respectively. Studies on the relationship between beer and immunity can be found in the supplementary data (Table 1).

**Table 1:** Studies related to the immunomodulatory effects of beer.

Classification	Aims of the study	Tested Parameters	Conclusions	References
Conventional beer	Moderate beer consumption, chronic disease.	J-shaped relationship between alcohol consumption.	Moderate beer consumption is associated with a lower incidence of cardiovascular events.	(16)
	Hops-derived compounds, adipocyte metabolism and glucose tolerance.	Insulin sensitivity markers Plasma levels.	Positive effects of META060 treatment and support the need to study such compounds in patients with obesity and type 2 diabetes.	(19)
	Beer components, cancer prevention potential.	Prenylated flavonoids.	Summarizes the chemo preventive activity of these compounds in relation to the inhibition of carcinogenic effects during the initiation, promotion, and progression phases, as well as the results of *in vivo* studies on metabolism, bioavailability, and efficacy.	(20)
	Potential benefits of beer in neurodegenerative diseases.	Cell viability and gene expression and protein levels of adenosine A1, A2A, A2B and A3 receptors.	Different beer extracts modulate adenosine receptors to different degrees.	(21)
	Short-term consumption of freeze-dried beer (LB), normotensive (WKY), hypertensive (SHR) rats.	Body weight, heart rate, systolic blood pressure, accumulation of GABA in the hypothalamus and brain medulla.	LB contains many bioactive compounds with a high antioxidant potential.	(22)
	Xanthohumol (XN) intake, wound healing.	Serum VEGF levels, N-acetylglucosaminidase activity, IL1β concentration, NO release.	XN intake can reduce inflammation, oxidative stress and angiogenesis and improve the wound healing process.	(23)
	Phenol-rich beverage consumption, wound healing angiogenesis, inflammation, oxidative stress regulation, diabetic rats.	Wounded skin micro vessel density, serum VEGF-A and inflammatory markers systemic glutathione, renal and hepatic H_2_O_2_, 3-nitrotyrosine and protein carbonylation.	Consumption of xanthohumol in diabetic animals consistently reduces inflammation and oxidative stress.	(24)
	Total phenols, flavanols and flavonoids extracted from beer samples were measured and their quality as antioxidants.	Total cholesterol, LDL cholesterol, HDL cholesterol, triglycerides, plasma albumin and fibrinogen, and antioxidant activity.	Short-term beer consumption had a positive effect on plasma lipid levels, plasma antioxidant and anticoagulant activity.	(25)
	Whether ethanol regulates the intracellular processes involved in IL-1β secretion was examined in cultured human macrophages.	Production of mature IL-1β.	Ethanol-induced inhibition of NLRP3 inflammasome activation in macrophages may represent a protective effect of moderate alcohol consumption against coronary artery disease.	(26)
Non-alcoholic beer	Cardiovascular effects, non-alcoholic components in beer.	Ethanol and phenolic compounds in beer.	Moderate alcohol consumption exerts cardioprotective effects.	(27)
	Beer consumption on CV risk.	Endothelial function, aortic stiffness, pressure wave reflection and aortic pressure.	In healthy non-smokers, beer acutely improves parameters of arterial function and structure.	(28)
	Both raw and freeze-dried wine and beer provided beneficial lipid and antioxidant effects in rats.	Dry red wine and Maccabee beer samples.	The bioactive compounds in these beverages appear to contain high levels of polyphenols.	(29)
	Moderate beer intake, metabolic syndrome.	Changes in body weight, lipoproteins, and vascular endothelial function.	Moderate beer intake may prevent lipid deposition in blood vessel walls.	(30)
	The cardiovascular protective, alcoholic beverages, polyphenols.	Number of circulating EPCs and EPC mobilizing factors.	Non-alcoholic beer increases the number of circulating EPCs in the peripheral blood of high-risk subjects.	(31)
	The diet of breastfeeding mothers with non-alcoholic beer, oxidative status, antioxidant content of their breast milk.	The oxidative status of mothers’ breast milk, plasma and urine and infants’ urine.	The consumption of non-alcoholic beer appears to improve the antioxidant capacity of breast milk and reduce oxidative damage in breastfeeding mothers.	(32)

### 2.1. The immunomodulatory power of conventional beer

Epidemiological studies have shown an association between moderate intake of fermented beverages and lower levels of inflammatory biomarkers, which is mainly attributed to the polyphenols, antioxidants, vitamins, and alcohol that are present in the beverages (33, 34). The polyphenolic component of beer interferes with pro-inflammatory pathways and induces inhibition of transcription factors or macromolecular complexes, thus reducing the synthesis and release of pro-inflammatory cytokines such as interleukins (IL) IL-1β, IL-6, IL-8, and tumor necrosis factor (TNF) (35). Studies have shown that moderate beer consumption increased IgG, IgM, and IgA concentrations as well as IL-2, IL-4, IL-10, and interferon (IFN)-γ production, resulting in decreased IFN-γ/IL-10 ratio in men and women, suggesting that moderate beer intake can produce the immunomodulatory effect (36).

The stimulation effect of low concentrations of ethanol on cellular immune responses was noted in the delayed cutaneous hypersensitivity-like responses evaluation (37). It has been noted that the intake of moderate amounts of alcohol is associated with a reduced risk of developing rhinovirus and the common cold (38). A complete immune response cannot be achieved without phagocytosis and the oxidative burst function of leukocytes. It has been noted that after 30 days of moderate beer consumption, there was a significant increase in the oxidative capacity of both neutrophils and monocytes (39). It can be speculated that moderate beer consumption may be associated with an increase in first-line immunity. In addition to those results, an *in vitro* study suggested that the beneficial effects on health may be related to the ability of beer to interfere with the pro-inflammatory cytokine cascade (40). It has even been suggested that this beverage has a dual anti-inflammatory effect of increasing IL-10 and attenuating the monocyte inflammatory response (41).

The abundant polyphenols in beer can exhibit a wide range of anti-inflammatory, antioxidant, and anti-aggregation activities mediated by the gut microbiome (36, 42). In mouse models of stress and depression, polyphenols can reduce the neuroinflammatory state by inhibiting the firing of neuronal cells (43).

Xanthohumol (XN) in beer inhibits the activity of inducible nitric oxide synthase and thus exerts anti-inflammatory effects. In addition, XN and humulone can produce anti-inflammatory effects by inhibiting TNF-α-induced endogenous synthesis of prostaglandin E2 by cyclooxygenase 2 (20, 44). It has been observed that the ingestion of XN via a natural beer matrix improves the wound-healing process in rats. This suggests that XN plays a role in reducing inflammation and oxidative stress, and in promoting angiogenesis (45).

Animal model studies have shown that isohumulone, a bitter substance in hops, can inhibit atherosclerosis. When ApoE-deficient mice (suffering from significant hyperlipidemia and progressive atherosclerotic lesions) were treated with isohumulone for 10 weeks, the area of atherosclerotic lesions in the aortic arch and aortic valve was significantly smaller than in controls. A significant decrease in serum IL-6 levels was also observed, suggesting that isohumulone slows the progression of atherosclerosis by inhibiting the inflammatory response during the atherosclerotic process (46). In addition, studies in hamsters noted that consumption of light or dark beer significantly inhibited atherosclerosis and reduced cholesterol and triglyceride levels (20). This suggests that moderate consumption of beer induces positive biochemical changes in blood composition that contribute to improving and preventing atherosclerosis.

**Figure 2:**
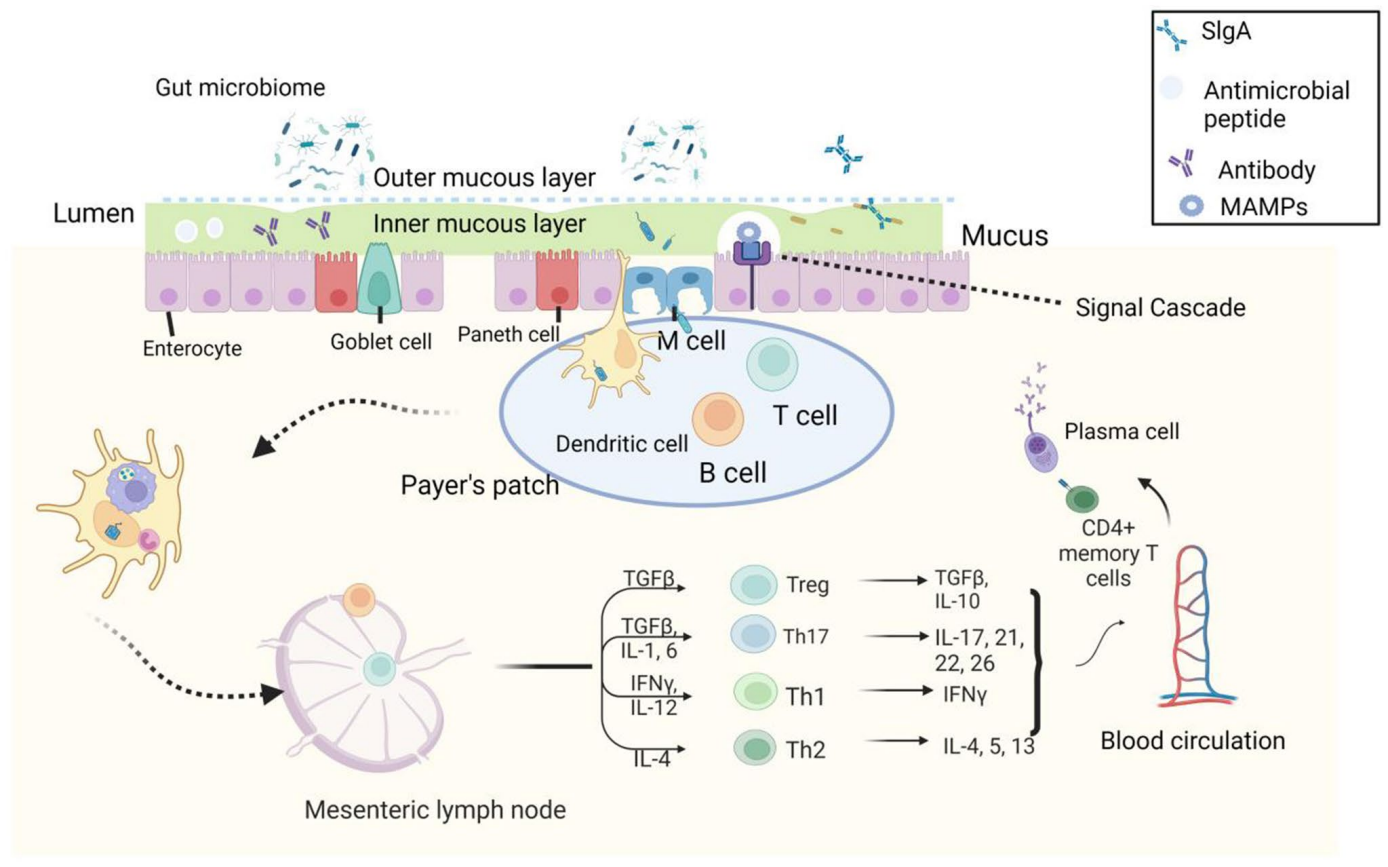
Microbial-associated molecular patterns (MAMPs) produced by commensal microorganisms stimulate Pattern recognition receptors (PRRs) in epithelial cells and induce the production of antimicrobial peptides. A few microorganisms are endocytosed into Peyer’s patches by M and DC cells, affecting the production of pro-inflammatory cytokines by intestinal epithelial cells (IECs) and dendritic cells (DCs) and macrophages present in the lamina propria (GALT) and Peyer’s patches. These cytokines such as thymic stromal lymphopoietin (TSLP), transforming growth factor (TGF), and interleukin-10 (IL-10) further recruit T and B cells, drive the production of mature, isolated lymphoid follicles, and release plasma cells that produce lgA. lgA binds to plgR to form a dimer that reaches the inner mucosa from the lamina propria after cleavage of plgR protein to release lgA, allowing it to exert its antimicrobial effect. Antigenic components obtained by a transmembrane sampling of DC cells can drain through the lymphatics to the mesenteric lymph nodes, inducing expansion of T cells and regulation of Th-1, Th-2, and Th-3 cell numbers (49–56).

Combining these results of studies in humans and animals, there is a consensus that moderate beer consumption has a beneficial effect on the immune system compared to states of alcohol abuse or abstinence (47, 48) (Figure 2).

### 2.2. Immunologic effectiveness of non-alcoholic beer

Several studies have shown that moderate beer intake can reduce the expression of inflammasome signaling pathways in human macrophages. In such studies, the normal beer groups had significantly reduced intracellular protein levels of pro-IL-1β in primed macrophages, and the release of cleaved IL-1β protein was blocked. Transcription of pro-inflammatory interleukins such as IL-1β and TNF-α was also significantly reduced in the non-alcoholic beer group (57). That is, non-alcoholic beer may also enhance the immune response, leading to a more effective defense.

A 45-day study of postmenopausal women consuming 500 mL of non-alcoholic beer per day found that intake of non-alcoholic beer lowered cholesterol levels in subjects with blood cholesterol greater than 240 mg/dL (58), which supports the role of long-term non-alcoholic beer consumption in combating mild chronic inflammation and preventing metabolic disorders. Similarly, supplementing mothers’ diets with non-alcoholic beer reduced oxidative damage in lactating mothers, thereby increasing the antioxidant capacity of breast milk (32).

In several studies in animals and humans, physiological stress resulting from prolonged high-intensity exercise was found to be associated with transient inflammation, immune dysfunction, and increased incidence of upper respiratory tract disease compared to moderate physical activity (59–63). In a study on healthy male runners, subjects consumed non-alcoholic beer daily for 3 weeks before and 2 weeks after the marathon. There was a reduction in IL-6 and total white blood cell count, and upper respiratory tract disease 24 h post-race. This positive result may be attributed to the polyphenolic compounds in non-alcoholic beer, which have antioxidant, anti-pathogenic, and anti-inflammatory properties (33).

## 3. Microbiota-mediated immunomodulatory efficacy of beer and its molecular mechanisms

The consumption of alcohol, as part of most people’s habits, has been controversial in terms of its effects on human health. If consumed inappropriately or in excess, it can trigger toxic reactions and social health burdens.

Alcohol causes dysbiosis of the gut microbiota in rodents and humans, as well as decreased production of beneficial metabolites such as short-chain fatty acids (SCFAs) (64, 65). Alcohol contributes to increased intestinal permeability and the transfer of bacterial products from Gram-negative microorganisms by enhancing oxidative stress in mitochondria (66) and disrupting the tight junctions of intestinal epithelial cells (67). The reflux of these toxic substances into the liver through the portal vein induces hepatic pathological damage such as alcoholic liver disease (ALD) (68). In addition, ethanol and its toxic metabolite acetaldehyde trigger intestinal barrier dysfunction, which can induce endotoxemia and systemic inflammation in the brain, intestine, and pancreas (69).

However, when alcohol consumption is controlled within safe limits, the combined effects of alcohol and other component metabolism on the intestinal flora deserve a more comprehensive analysis (70).

It is well known that the gut microbiome uses diet as a bridge to form a complex and dynamic mutually beneficial symbiosis with the host. At the same time, the intestinal mucosa and its vast micro-ecosystem together form a complex and dynamic immune barrier that participates in disease prevention and regulation of immune function through the interaction of microorganisms with the immune system (71).

Most of the water, vitamins, and other low molecular weight substances in beer are absorbed in the stomach and small intestine. Polyphenols and other important substances reach the colon, where they meet the intestinal microbiota and are eventually transformed (72, 73). Their metabolites inhibit pathogenic bacteria, stimulate the proliferation and activity of healthy flora such as *Lactobacilli* and *Bifidobacteria*, and regulate the intestinal microbiota (74). In other words, the polyphenols in beer have prebiotic properties (75).

### 3.1. Human studies

Studies have shown that phenolic substrates and the resulting aromatic metabolites supplied to intestinal bacteria through dietary intake may in turn cause fluctuations in microbial community composition through selective prebiotic effects and modulation of antimicrobial activity against enteropathogenic bacteria (76–78).

It has been noted that aromatic metabolites produced by the metabolism of epicatechin, catechin, 3-O-methylmalonic acid, and caffeic acid in beer significantly inhibit the growth of pathogenic bacteria such as *Clostridium perfringens* and *Clostridium difficile* without affecting the growth of *Bifidobacterium* (75). Flavonols in beer induce the growth of *Lactobacillus* and *Bifidobacterium* and may decrease plasma C-reactive protein concentrations, suggesting that beer may provide immunological benefits (79). In the human intestine, six species of *Streptococcus* are associated with immunomodulatory functions (80). An increase in the abundance of flora with immunomodulatory functions has been documented with the consumption of non-alcoholic and alcoholic beers: including *Streptococcus*, *Actinomyces*, *Veillonella*, *Bacillus*, *Lactococcus*, and *Weissella*. Among them, *Actinomyces* has important functions in the regulation of intestinal permeability, the immune system, metabolism, and the gut-brain axis. In an article on the effects of moderate beer consumption on human gut health (71), it was found that 30 days of non-alcoholic beer daily consumption resulted in increased numbers of *Streptococcus* spp., *Bacillus spp.*, and *Paramecium spp.* as well as changes in glucose metabolism, and lipopolysaccharide and phenylalanine synthesis in the intestine. Of interest is the fact that the phenylalanine synthesis pathway is associated with resveratrol production (80) which may be beneficial in the treatment of cancer, neurological diseases, cardiovascular diseases, and nonalcoholic fatty liver disease (81).

It is known that uropathogens can form biofilms that protect bacteria from the host immune responses and antimicrobial treatments, making the treatment of urinary tract infections a challenge. A study isolated *Lactobacillus fermentum* TIU19 from beer and showed that it may act as a potential probiotic against biofilm formation and inhibit the activity of the multi-drug resistant uropathogenic *E. coli* and *E. faecalis* (82). A similar study was conducted on *Vibrio vulnificus* DU14 isolated from traditional fermented rice beer from the Sivsagar district of Assam, which also has probiotic potential (83).

Intestinal microbiota act in coordination with the intestinal barrier, protecting the body from disease and stimulating the immune system (84). The production of short-chain fatty acids after polyphenol ingestion improves intestinal permeability, thus reducing intestinal inflammation and endotoxemia (85). In addition, short-chain fatty acids are key substrates of the cross-feeding system, which is a component of the intestinal flora. For example, cross-feeding occurs between *Bifidobacterium longum* and *Eubacterium rectale*, and both strains consume arabinoxylan, which is the main source of dietary fiber in beer. However, *Bifidobacterium longum* was additionally stimulated by consuming monosaccharides released by extracellular degradation of arabinoxylan by *Eubacterium rectale*, resulting in a reciprocal cross-feeding effect. In addition, *Faecalibacterium prausnitzii* can produce butyric acid from lactic acid produced by *Bifidobacteria*. In the presence of arabinoxylan, *Bifidobacteria* and butyric acid-producing colonic bacteria (*Clostridium praecalibacterium*, *Eubacterium rectum,* and *Roseburia* spp.) were stimulated simultaneously, leading to a significant increase in butyric acid production (13).

### 3.2. Animal studies

Ferulic acid is the most abundant polyphenol in beer (86). A study in mice showed that ferulic acid altered the composition of the intestinal microbiota by modulating the ratio of thick-walled to bacterial flora, making it closely associated with specific intestinal microbiota and the genetic regulation of triglyceride and total cholesterol metabolism in nonalcoholic fatty liver disease (87). Recent studies have reported that the gut microbiome can convert ellagic acid to urolithin (88). Urolithins can cross the blood–brain barrier (89) and may affect mice with Alzheimer’s disease by reducing neuronal inflammation (81). In addition, we found different conclusions in experiments using barley beer waste pellets fed to rats (90) and in experiments analyzing fecal samples from abstainers and moderate drinkers (13). Both studies showed that the intake of beer modulates the levels of butyric acid, which then interacts directly with the host’s immune system and reduces the levels of inflammatory markers (14).

We have reason to believe that beer, mediated by the gut microbiome (20), exerts beneficial immunomodulatory effects in humans. Studies on the relationship between beer, the gut microbiome, and immunity can be found in the supplementary data (Table 2) (96, 97).

**Table 2:** Study on the relationship between immune effect of beer and gut microbiome.

Classification	Aims of the study	Tested parameters	Observations	Ethanol references
Human research	Moderate beer consumption, microbiota composition， short-chain fatty acid (SCFA) profile.	Gut microbiota composition，SCFA concentration.	Moderate beer consumption has potentially beneficial effects on intestinal health.	(13)
	The ability of gordonia bacteria, anti-inflammatory urolithin.	The excretion of iso-urolithin-A and/or urolithin-B	The beneficial effects of eating foods containing ellagic acid may be mediated by an individual’s level of Gordonia.	(88)
	Supercritical CO2 extracts of hops, intestinal bacterial	Microbial metabolism was assessed by organic acid production	Hop compounds have a significant effect on the structure of bacteria.	(91)
	Pomegranate extract, miRs expression in surgical colon tissues of CRC patients.	Changes of urolithin and miRs	There were significant differences in specific miRs between malignant and normal tissues.	(92)
	Effects of moderate beer consumption, human health, intestinal microflora function.	Blood samples and stool samples.	Moderate intake of NAB can have a positive impact on human health by supplementing bioactive polyphenols and phenolic acids and enriching the diversity of intestinal flora with beneficial bacteria.	(93)
	Phenylacetic acid (PAA),4-hydroxyphenylacetic acid (4-hydroxyYPAA), microbial fermentation of aromatic amino acids (AAAs) in the colon.	Phenylalanine, tyrosine, and tryptophan.	Certain microbial species can ferment all three AAAs, and that protein fermentation may be the source of major phenylpropane-derived metabolites in the colon.	(94)
Animal research	Effect of phenolic components of a tea extract.	Faecal homogenates containing bacteria had a significant catalytic effect on the aromatic metabolites.	The tea phenolics exerted a significant influence on the intestinal environment.	(74)
	Ferulic acid (FA) on non-alcoholic fatty liver disease, intestinal microflora.	Liver morphology, lipid profile, intestinal microflora	FA has the potential to improve NAFLD.	(87)
	Metabolic effects of cranberry.	The composition of intestinal flora.	CE treatment can reduce hfHS-induced weight gain and visceral obesity.	(90)
	The mechanism, metabolic syndrome, intestinal flora.	Serum inflammatory markers.	Intestinal flora may provide a missing link in the mechanism of dietary polyphenol malabsorption.	(95)
	Metabolic effects of pitaya fruit β anthocyanidin (HPBN), high-fat diet mice, regulation of intestinal flora.	The composition of intestinal flora.	HPBN has clinical significance in the treatment of obesity, non-alcoholic fatty liver disease and type 2 diabetes.	(96)
	Resveratrol is produced biologically by genetic engineering of several microbial hosts.	Resveratrol.	In recent years, the research progress of resveratrol synthesis by engineering bacteria was reviewed, with emphasis on metabolic engineering modification and optimization of production process.	(57)

## 4. Discussion

Beer is a long-established alcoholic beverage that is rich in a variety of nutrients and micronutrients. This review focuses specifically on the interactions and mechanisms between beer and the gut microbiome in the regulation of body immunity.

The risk of death is lower in light and moderate drinkers and increased in heavy drinkers. Previous evidence from the literature on animal experiments (87–90, 96, 97), and human experiments (33–39) suggests that low or moderate beer consumption, with or without alcohol, shows positive health effects by stimulating the development of a healthy microbiota. In particular, it has been shown to be effective in reducing the incidence of coronary heart disease, Alzheimer’s disease, metabolic syndrome, cancer, and Type-2 diabetes. Disease-related indicators have also been noted including reductions in fibrinogen (anticoagulation) and C-reactive protein (anti-inflammatory); and increases in HDL (anti-atherosclerotic) and adiponectin (improves glucose homeostasis) levels (5).

The gut microbiome has long played an important role in human health. This review demonstrates the environment-diet-microbial-host interactions through Figure 1. It is clear from the figure that when beer is consumed in moderation, the phenols and other nutrients it contains are fermented and broken down by the microbial community that resides in the outer mucosal layer of the gut. This miraculous digestive process produces a large number of metabolites that, through the interaction of multiple microorganisms in the inner mucosa, in turn, promote changes in the abundance of beneficial flora, exerting a range of anti-inflammatory, antioxidant, and immunomodulatory effects. In this virtuous cycle, the gut microbiota provides a platform for information exchange. We demonstrate the mechanism by which these fermentation breakdown products function on this platform in Figure 2 (49–56): fermentation products promote the growth of beneficial flora, and microbial-associated molecular patterns (MAMPs) produced by these microbes stimulate pattern recognition receptors (PRRs) in epithelial cells and induce the production of antimicrobial peptides. A few are endocytosed into Peyer’s patches by M and dendritic cells (DCs) cells, which produce pro-inflammatory cytokines or chemokines (thymic stromal lymphopoietin (TSLP), transforming growth factor (TGF) and interleukin-10 (IL-10)) that act on effector cells and induce maturation of lymphoid follicles and release of lgA. antigenic components acquired by DC cells can drain through the lymphatic vessels to the mesenteric lymph nodes, induce the expansion of T cells, regulate the number of Th-1, Th-2, and Th-3 cells, and initiate the mucosal immune barrier.

Evidence from the literature (16, 19–32) suggests that the immunomodulatory effects of beer are attributed to the three components it contains: polyphenols, fiber, and ethanol. Due to the conversion of beer substrates, the formation of bioactive end products, and the presence of microorganisms, some of its components exert “similar” or even greater effects than probiotics. Polyphenols and fiber show the potential to enhance the development of a healthy gut microbiome through probiotic mechanisms, dominated by sugar-catabolizing, short-chain fatty acid-producing bacteria (*Bifidobacterium* spp. and *Saccharomyces* spp.). Polyphenols and picric acid constitute the antioxidants of beer, exerting a counteracting effect against intestinal flora dysbiosis and local anti-inflammatory effects. Beer contains dietary fibers such as non-starchy, non-digestible carbohydrates (β-glucan, arabinoxylan, mannose, fructose polymers, etc.) that, through fermentation, establish contact with the intestinal microbiota and act as nutritional substrates to stimulate the development of the gut microbiome. Most of all these reported studies assessed the effect of the above substances on microbiota by monitoring changes in SCFAs. In addition, the acetate produced by alcohol may stimulate energy metabolism and prevent cardiovascular disease.

To date, the mechanisms of interaction between beer and the gut microbiome in immunomodulation have not been adequately studied, and the available results are based on inference rather than quantification of specific effects. Low or non-alcoholic beers are good candidates for functional foods. Health beers made by fortifying them with bioactive substances such as fiber, antioxidants, and probiotics would provide health benefits to consumers. Whether beer can be used in the future as a micro-ecological regulator or even as an alternative therapy for chronic diseases such as hypertension, diabetes, and obesity is a question that deserves further research.

## Author contributions

HL: conceptualization and modification. SZ: manuscript writing, figure drawing, and modification. SJ: manuscript writing. CZ and SH: modification. All authors contributed to the article and approved the submitted version.

## Funding

This study was supported by the Open Research Fund of State Key Laboratory of Biological Fermentation Engineering of Beer, under grant no. K202101.

## Conflict of interest

CZ and SH were employed by Tsingtao Brewery Co. Ltd.

The remaining authors declare that the research was conducted in the absence of any commercial or financial relationships that could be construed as a potential conflict of interest.

## Publisher’s note

All claims expressed in this article are solely those of the authors and do not necessarily represent those of their affiliated organizations, or those of the publisher, the editors and the reviewers. Any product that may be evaluated in this article, or claim that may be made by its manufacturer, is not guaranteed or endorsed by the publisher.
